# Clinical and pathological features of aspergillosis due to *Aspergillus fumigatus* in broilers

**DOI:** 10.14202/vetworld.2020.2787-2792

**Published:** 2020-12-26

**Authors:** Alfarisa Nururrozi, Yanuartono Yanuartono, Sitarina Widyarini, Dhasia Ramandani, Soedarmanto Indarjulianto

**Affiliations:** 1 Department of Internal Medicine, Faculty of Veterinary Medicine, Universitas Gadjah Mada, Yogyakarta, Indonesia; 2 Department of Pathology, Faculty of Veterinary Medicine, Universitas Gadjah Mada, Yogyakarta, Indonesia; 3 Department of Bioresource and Veterinary Technology, Vocational College, Universitas Gadjah Mada, Yogyakarta, Indonesia

**Keywords:** aspergillosis, broiler, clinical, pathological

## Abstract

**Background and Aim:**

*Aspergillus fumigatus* is a ubiquitous pathogen causing aspergillosis in poultry. This research aimed to evaluate the clinical and pathological features of aspergillosis infection in broilers.

**Materials and Methods:**

*A. fumigatus* infection was induced experimentally by intra-air sac inoculation of a 1.7×10^8^ spore suspension into broilers. Infected and non-infected birds were closely observed for the development of clinical signs of infection twice daily. Pathological samples were collected 5, 14, and 30 days post-infection (dpi) and examined by hematoxylin-eosin staining.

**Results:**

A total of 160 birds were included in this study. Clinical signs emerged at 3 dpi and became consistent at 5 dpi. A considerable decrease in severity and number of birds showing infection symptoms followed. The clinical signs of aspergillosis included anorexia (n=40; 50%), lethargy (n=32; 40%), dyspnea (n=38; 48%), and gasping (n=29; 36%). Macroscopic changes in the air sacs at 3 dpi included the development of minor lesions showing cloudiness, slight membrane thickening, and local exudates. Histopathological examination of the air sacs collected at 3 dpi indicated local inflammation surrounded by hyphae and spores. At 5 dpi, infected birds developed nodules, necrosis, and parenchymal consolidation of the lungs. Pulmonary changes, such as bronchopneumonia, spores, septate hyphae, and mild granulomatous inflammation, were also observed. At 14 dpi, multiple caseous nodules and plaques were found in the air sacs; plaque and necrosis in large areas of the lungs and severe multifocal granulomatous inflammation were noted.

**Conclusion:**

The clinical symptoms of aspergillosis emerged at 3 dpi and gradually decreased beginning at 7 dpi. Similar pathological changes were observed in the air sacs and lungs. The results of this work provide additional information on the pathogenesis of aspergillosis.

## Introduction

Aspergillosis is the major mycotic disease in birds [1]. *Aspergillus fumigatus* have been reported as the most frequently isolated pathogen [2]. *A. fumigatus* can infect nearly all types of birds, including poultry and pets [3,4]. Acute aspergillosis of young birds (i.e., aged 3 days-20 weeks) may result in mortality rates between 4.5% and 90% and cause great economic losses [2]. Aspergillosis can cause direct loss through the death of birds, impaired growth-feed conversion, and immunosuppressive effects [1,5]. Although aspergillosis caused by *A. fumigatus* is a pathogen, some *aspergillus* also has been used for dietary supplementation in the birds. *Aspergillus niger* improved growth performance and meat quality [6], *Aspergillus awamori* provided the alternative of probiotic and antibiotics effect [7], and combination *A. awamori*-lactic acid bacteria increasing unsaturated fatty acid and reducing saturated fatty acid on egg yolk [8]. *A. fumigatus* shows optimal growth at the normal body temperature of birds (40-42°C); however, the spores can also grow well at 37-38°C [9].

Research on aspergillosis in broiler chickens in Indonesia is limited despite the number of cases reported by farmers annually and the significant economic impact of the disease. Observation of clinical signs may be a rational approach to determine the diagnosis and treatment of aspergillosis [10,11]. Late diagnosis and treatment failure often results in poor prognosis [2,5]. Animal models offer an alternative approach for aspergillosis studies [12-14]. Research by artificial infection is likely to provide valuable information regarding the pathogenesis of aspergillosis.

The pathophysiology of the disease caused by *A. fumigatus* isolates obtained from Indonesia has not been fully understood. Therefore, this study aimed to evaluate the clinical and pathological features of experimental aspergillosis in broilers. The results of this work provide basic data for determining rational actions in response to aspergillosis infection.

## Materials and Methods

### Ethical approval

This research was carried out after procuring the necessary approval from the Ethical Clearance Commission for Preclinical Research of Laboratory Research and Integrated Testing, Universitas Gadjah Mada, Indonesia (No. 233/KEC-LPPT/III/2015).

### Study period and location

This research was conducted for 4 months (March to June 2015), consisting of pre-research, experimental, and laboratory examination. The birds were reared in the experimental facilities, Faculty of Veterinary Medicine, Universitas Gadjah Mada. Laboratory examinations were conducted at the Department of Internal Medicine and the Department of Pathology.

### Animals

A total of 160-day-old male Lohmann broilers (reared at experimental facilities of the University) were randomly divided into two groups, including the infected group and the control group. The birds were reared under strict hygienic conditions for 30 days and housed separately in different cages. Sterilized commercial poultry mash and water were provided *ad libitum*.

### Experimental inoculum

The *A. fumigatus* strain used in this research was obtained from The Food and Nutrition Culture Collection, Center of Food and Nutrition Study, Universitas Gadjah Mada, Indonesia. The strain was isolated from a bird in Indonesia suffering from aspergillosis. *A. fumigatus* was cultured on Sabouraud dextrose agar (HiMedia, India) supplemented with chloramphenicol (0.5 g/L Indofarma, Indonesia) for 3-4 days at 37°C. *A. fumigatus* appears on the agar as raised clumps that are green or bluish-gray in color. After 3 days of incubation, conidia were produced from phialides at the ends of conidiophores. These conidia were harvested by flushing the plates with normal saline solution (NSS) and then washed with phosphate buffer saline. The tube containing the conidium suspension was then shaken by a mechanical shaker to break down the conidia. Spores were quantified using a hemocytometer (Heinz, Germany).

### Standardization of infective dose

A pre-research using 200-day-old chicks randomly divided into five groups (n=40) was conducted to determine the LD50. Each bird was inoculated by intra-air sac injection with a spore suspension equivalent to 1×10^8^, 4×10^8^, 8×10^8^, and 12×10^8^ spores per 0.1 mL of NSS. The chicks in the fifth group were kept as the control group and inoculated with 0.1 mL of NSS. All birds were observed for clinical signs and mortality up to 30 days. The accumulated mortality was calculated in all groups, and the data were used to determine LD50 according to the method of Reed and Muench. The LD_50_ was determined to be 1.7×10^8^ spores per 0.1 mL; thus, this dose was employed in subsequent experiments.

### Experimental design

*A. fumigatus* was induced experimentally on day 1 by intra-air sac inoculation of a spore suspension. Each bird in the infected group was administered 0.1 mL of NSS containing 1.7×10^8^ spores. The control group was similarly injected with 0.1 mL NSS without spores. The development of the clinical signs of infection was observed twice daily, and the appearance of clinical signs or mortality was recorded. Necropsy and re-isolation of the fungus were carried out on all dead chickens. Pathological samples were collected on days 3, 5, and 14 through the sacrifice of five birds on each observation day. All organs were fixed in 10% formaldehyde and stained with hematoxylin-eosin.

### Pathological examination

The severity of infection was scored on the basis of macroscopic and microscopic changes, as shown in Tables-1 and 2, respectively. The degree of scoring used in this work was modified from a scoring method reported in the previous research [10,15].

**Table-1:** Scoring of macroscopic changes in broiler organs.

Score	Characteristics
0	• No macroscopic changes were found in the respiratory system organs
1	•Cloudy air sac, slight thickening, and local exudate
	• Edema and hyperemic lungs; focal necrosis
2	• Yellow caseous exudate covering the air sac
	• Nodules and necrosis in over half of the lung area, parenchymal consolidation
3	• Multiple caseous nodules or plaques, severe thickening of the air sac
	• Nodule/plaque formation and necrosis in wide areas of the lung, massive congestion
	• Growth of fungal colonies in other respiratory organs or visceral organs

**Table-2:** Scoring of microscopic changes in broiler organs.

Score	Characteristics
0	•No microscopic changes were found in the respiratory system organs
1	• Local inflammation
2	• Hyphae/spores/conidiospores found
3	• Hyphae/spores/conidiospores found
	• Mild granulomatous inflammation
4	• Hyphae/spores/conidiospores found
	•Granulomatous inflammation (>2 in each field of view)
	• Giant cells and severe necrotic cells

### Statistical analysis

Data of the clinical and pathological features of the broilers were analyzed descriptively.

## Results

### Clinical signs

The clinical signs of aspergillosis began to emerge 3 days post-infection (dpi). The experimentally infected birds showed anorexia, lethargy, dyspnea, and gasping. Specifically, the chickens initially showed dyspnea and gasping, followed by anorexia, which caused them to become weak. The control group showed no clinical signs on the same day of observation.

**Figure-1:**
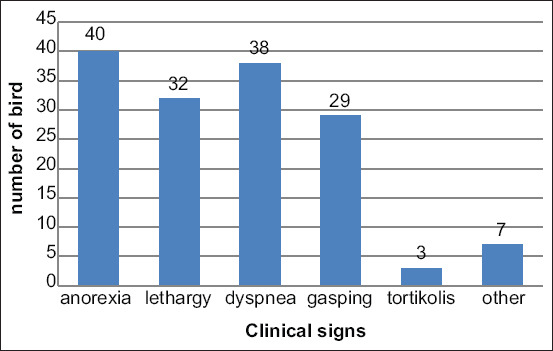
Clinical observations of *Aspergillus fumigatus* infection.

Clinical signs were observed in a varying number of birds from 3 dpi to 8 dpi. The number of birds showing clinical signs, as well as the severity of symptoms, began to decrease at 8 dpi and then completely disappeared at 12 dpi. The clinical signs of aspergillosis included anorexia (n=40; 50%), lethargy (n=32; 40%), dyspnea (n=38; 48%), and gasping (n=29; 36%) (Figure-1). Some sporadic birds showed symptoms of other clinical signs, including torticollis, conjunctivitis, ascites, and stunting.

According to Okwara [16], the symptoms of aspergillosis primarily consist of increased heavy breathing and a scratchy sound heard chiefly during expiration. The clinical signs of dyspnea gradually become more severe, and snoring develops. The bird then loses its appetite, tends to ruffle its feathers, and becomes sleepy.

**Figure-2:**
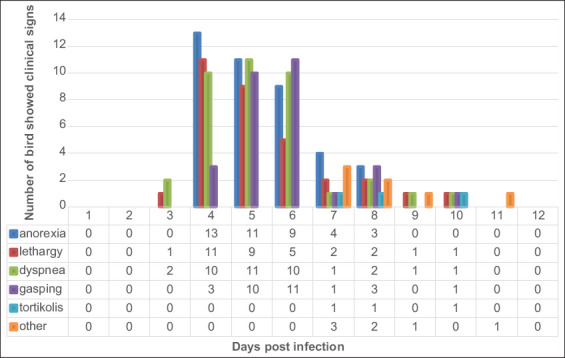
Clinical signs of aspergillosis from 2 days to 11 days post-infection.

The infected group did not show clinical symptoms of aspergillosis until 2 dpi. At 4 dpi, 37 of the 80 infected chickens began to show clinical signs of aspergillosis. Control group chickens injected with physiological NaCl showed no clinical symptoms on the same day. At 5 dpi, 41 sick chickens were recorded. At 6 dpi, the number of chickens showing clinical signs of aspergillosis gradually decreased (Figure-2).

### Macroscopic lesions

Necropsy was carried out on all dead chickens for macroscopic and microscopic pathological observation. The data of pathological changes recorded at different time points are shown in Tables-3 and 4.

**Table-3:** Macroscopic lesions in the infected group.

Organ	Air sacs				Lungs			
		
dpi/score	0	1	2	3	0	1	2	3
3	1/5	3/5	1/5		4/5		1/5	
5			3/5	2/5		1/5	3/5	1/5
14			1/5	4/5			2/5	3/5

**Table-4:** Microscopic lesions in the infected group.

Organ	Air sacs					Lungs				
		
dpi/score	0	1	2	3	4	0	1	2	3	4
3		1/3	2/3			1/3	2/3		
5		1/3		2/3				3/3		
14					3/3				2/3	1/3

## Discussion

The clinical signs of early-onset aspergillosis observed in infected birds were breathing difficulties and increased frequency of inhalation; birds infected with aspergillosis often breathed by craning their neck or opening their beak. Respiratory problems appeared to be more common at night than during the day, possibly because of a limited oxygen supply. Anorexia levels in infected birds ranged from mild to severe. These findings are consistent with the previous research [13,17,18], which reports that the clinical symptoms of acute aspergillosis manifest from between few days to 2 weeks of early maintenance. Aspergillosis has been clinically discovered in young chickens aged <13 days [19].

Dyspnea and gasping caused by hyphae growth lead to necrosis and inflammation in the air sacs and lungs [20]. Necrotic cells in the respiratory tract cause hypoxia, and the birds compensate for the oxygen demand of tissues by increasing their respiratory frequency. Exudates and inflammation in the lung cause disrupted air circulation [21]. The presence of plaques or necrotic areas in the respiratory tract due to aspergillosis could inhibit the exchange of oxygen in the lungs [2].

In this study, intra-air sac infection with 1.7×10^8^ spores caused acute aspergillosis with a morbidity rate of 71.25%, thus confirming the data of earlier reports [11,16,20]. Mortality occurred primarily within the first 5 days after infection.

**Figure-3:**
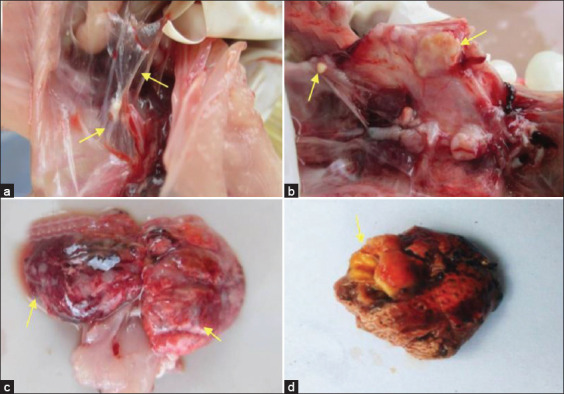
Macroscopic lesions in the infected group. (a) Thickening and turbidity of the walls of the thoracic air sac (3 dpi). (b) Multiple caseous nodule or plaques and severe thickening of the air sacs (14 dpi). (c) Nodules and necrosis in the lungs (5 dpi). (d) Caseous plaques in wide areas of the lung (14 dpi).

Macroscopic changes in the lungs of infected birds at 3 dpi included minor lesions (Table-5, Figure-3). A few birds revealed caseous nodules, necrosis in over half of the lung area, and parenchymal consolidation. The 3 dpi has not been found in the peripheral edema and pulmonary parenchymal consolidation. The results of this study support the previous publication by Cheng *et al*. [15], which states early in the pulmonary lesions occur on the edges of pulmonary edema, progressive consolidation, and formed small white nodules.

**Table-5:** Most prominent macroscopic changes observed in broilers infected with aspergillosis.

Days post-infection	Macroscopic lesion	

	Air sac	Lung
3	Cloudy, slightly thickened, local exudate	No macroscopic changes were found
5	Yellow caseous exudate covering the air sac	Nodules and necrosis in over half of the lung area, parenchymal consolidation
14	Multiple caseous nodules or plaques, severely thickened air sac	Nodules/plaques and necrosis in wide areas of the lung, massive congestion

At 5 dpi, the infected birds showed severe lung lesions (Table-5, Figure-3); indeed, nearly all of the birds in the infected group showed macroscopic lesions. Yellowish-white caseous nodules diffused throughout the lung to form plaque aggregates with a diameter of 5-8 mm spread evenly over the tissue. Large necrotic areas and parenchymal consolidation were found in the lungs at 5 dpi.

**Figure-4:**
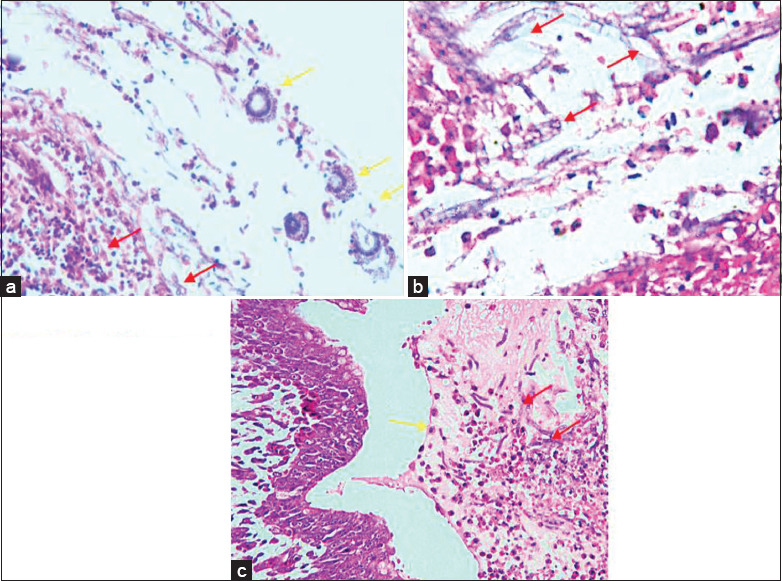
(a) Local inflammation surrounded with hyphae and spores in air sacs at 3 dpi. (b) Granulomatous inflammation surrounded hyphae, submucosal edema, and infiltration of inflammatory cells at 5 dpi. (c) Bronchopneumonia with spores and septate hyphae at 5 dpi.

Examination of air sacs at 3 dpi revealed local inflammation surrounded with hyphae and spores (Table-6, Figure-4a). No granulomas were observed at 3 dpi. Several researchers [2,10,13-15] have found that microscopic changes in the air sacs of infected birds develop rapidly and are accompanied by thickening, vascularity, and turbidity. Granulomas measuring 1-5 mm appeared and tended to merge to form plaques. Caseous plaques in the air sacs formed from the merging of fungal colonies, eventually covering and blocking the entire lamina membrane [2,10,15].

**Table-6:** Most prominent microscopic changes observed in broilers infected with aspergillosis.

Days post-infection	Microscopic lesion	

	Air sac	Lung
3	Inflammation, hyphae, and spores found	Local inflammation
5	Granulomatous inflammation	Hyphae and spores found
14	Granulomatous inflammation (>2 in each field of view)	Granulomatous inflammation
	Giant and severenecrotic cells	

Microscopic examination of organs of the control group at 5 dpi revealed the absence of lesions (Table-6). The air sacs of this group appeared normal; no accumulation of inflammatory cells and/or changes in the epithelium was noted. Microscopic changes in the air sacs of chickens in the infected group at 5 dpi indicated granulomatous inflammation surrounded by spores and hyphae, submucosal edema, infiltration of inflammatory cells, and epithelial hypertrophy of the air sacs (Table-6, Figure-4b). Hypertrophy of epithelial cells in air sacs caused macroscopic changes leading to membrane thickening. Infiltration of mononuclear inflammatory cells dominated by heterophils was observed all over the air sacs. Spores were surrounded by inflammatory cells and cell debris. The absence of a submucosal layer, which indicates the occurrence of edema, was also noted.

The emergence of conidia and hyphae is closely related to the virulence factors of aspergillosis. *A. fumigatus* conidia are fairly small and have a diameter of only 2-3 μm. Thus, these conidia can pass through physical barriers and invade all tissues of the respiratory system [21,22]. *A. fumigatus* spores have intracellular germination capability, which is associated with the degeneration and necrosis of macrophages.

Microscopic lesions in the lungs of chickens in the infected group at 5 dpi revealed bronchopneumonia and infiltration of inflammatory cells dominated by heterophils in the bronchi and around vesicles. Pathognomonic microscopic findings in pulmonary were bronchopneumonia, spores, septate hyphae, and mild granulomatous inflammation (Table-6, Figure-4c).

Cacciutolo *et al*. [22] stated that the initial stage of aspergillosis infection is characterized by focal lymphocytes and macrophages. Caseous necrosis with the proliferation of connective tissue was observed at 4 dpi. Accumulation of caseous exudates in the bronchial lumen and air vesicles caused respiratory symptoms such as dyspnea, gasping, panting, and coughing.

**Figure-5:**
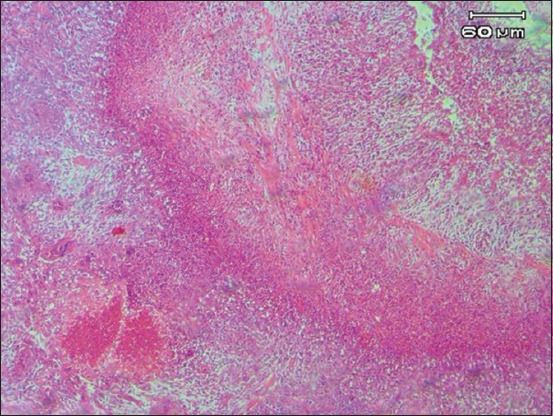
Granulomatous inflammation of the lungs with severe necrotic areas, heterophils surrounded by macrophages, and connective tissue.

Histopathological observations at 14 dpi showed no different results compared with 5 dpi (Table-6, Figure-5). Hyphae surrounded by severe multifocal granulomatous inflammations (i.e., >2 in a single field of view) were also found. Observation of all groups at 14 dpi revealed no spores (Figure-5). Lesions developed into granulomas consisting of severe necrotic areas and heterophils surrounded by macrophages, lymphocytes; connective tissues were also found [23].

## Conclusion

The clinical symptoms of aspergillosis decreased by 7 dpi. Pathological features indicated permanent organ damage. Birds affected by aspergillosis should be culled because of irreversible pathological damage.

## Authors’ Contributions

SI conceptualized, managed, and supervised the study. AN and DR collected, recorded, and analyzed the samples. YY and SW identified and analyzed data. All authors drafted, revised and approved the final manuscript.

## References

[1] Beernaert L.A, Pasmans F, Van Waeyenberghe L, Haesebrouck F (2010). *Aspergillus* infections in birds:A review. Avian Pathol.

[2] Arné P, Thierry S, Wang D, Deville M, Le Loc'h G, Desoutter A, Féménia F, Nieguitsila A, Huang W, Chermette R, Guillot J (2011). *Aspergillus fumigatus* in poultry. Int. J. Microbiol.

[3] Martin M.P, Bouck K.P, Helm J, Dykstra M.J (2007). Disseminated *Aspergillus flavus* infection in broiler breeder pullets. Avian Dis.

[4] Zafra R, Perez J, Perez R.A (2008). Concurrent aspergillosis and ascites with high mortality in a farm of growing broiler chickens. Avian Dis.

[5] Saif Y.M (2008). Diseases of Poultry.

[6] Saleh A.A, Eid Y.Z, Ebeid T.A, Kamizono T, Ohtsuka A, Hayashi K (2011). Effects of feeding *Aspergillus awamori* and *Aspergillus niger* on growth performance and meat quality in broiler chickens. J. Poult. Sci.

[7] Saleh A.A, Hayashi K, Ijiri D, Ohtsuka A (2014). Beneficial effects of *Aspergillus awamori* in broiler nutrition. Worlds Poult. Sci. J.

[8] Saleh A.A, Gálik B, Arpášová H, Capcarová M, Kalafová A, Šimko M, Juráček M, Rolinec M, Bíro D, Abudabos A.M (2017). Synergistic effect of feeding *Aspergillus awamori* and lactic acid bacteria on performance, egg traits, egg yolk cholesterol and fatty acid profile in laying hens. Ital. J. Anim. Sci.

[9] Munir M.T, Rehman Z.U, Shah M.A, Umar S (2019). Interaction of *Aspergillus fumigatus* with the respiratory system in poultry. Poult. Sci. J.

[10] Femenia F, Fontaine J.J, Lair-Fulleringer S (2007). Clinical, mycological and pathological findings in turkeys experimentally infected by *Aspergillus fumigatus*. Avian Pathol.

[11] Sultana S, Harun-Ur-Rashid S.M, Islam M.N, Ali M.Z (2014). Pathological investigation of avian aspergillosis in commercial broiler chicken at Chittagong district. Int. J. Adv. Res. Biol. Sci.

[12] Clemons K.V, Stevens D.A (2005). The contribution of animal models of aspergillosis to understanding pathogenesis, therapy and virulence. Med. Mycol. J.

[13] Beernaert L.A, Pasmans F, Haesebrouck F, Martel A (2008). Modelling *Aspergillus fumigatus* infections in racing pigeons (*Columba livia domestica*). Avian Pathol.

[14] Desoubeaux G, Cray C (2018). Animal models of aspergillosis. Comp. Med.

[15] Cheng Z, Li M, Wang Y, Chai T, Cai Y, Li N (2020). Pathogenicity and immune responses of *Aspergillus fumigatus* infection in chicken. Front. Vet. Sci.

[16] Okwara N (2016). Aspergillosis in Turkeys:A review. J. Agric. Vet. Sci.

[17] Singh S, Borah M.K, Sharma D.K (2009). Aspergillosis in Turkey poults. Indian J. Vet Pathol.

[18] Melo A.M, Silva-Filho R.P, Poester V.R, Von Groll A, Fernandes C.G, Stevens D.A, Sabino R, Xavier M.O (2020). Aspergillosis in free-ranging aquatic birds. Med. Mycol. Case Rep.

[19] Beernaert L.A, Pasmans F, Baert K (2009). Designing a treatment protocol with voriconazole to eliminate *Aspergillus fumigatus* from experimentally inoculated pigeons. Vet. Microbiol.

[20] Ahamad D.B, Ranganathan V, Punniyamurthy N, Sivaseelan S, Puvarajan B (2018). Pathomorfology of aspergillosis in a Japanese quail. Indian Vet. J.

[21] Chu J, Zhang Q, Zuo Z.H, Elashram S, Guo Y.X, Zhao P (2017). Co-infection of *Chlamydia psittaci* with H9N2, ORT and *Aspergillus fumigatus* contributes to severe pneumonia and high mortality in SPF chickensx. Sci. Rep.

[22] Cacciutolo E, Rossi G, Nardoni S, Legrottaglie R, Mani P (2009). Anatomopathological aspect of avian aspergillosis. Vet. Res. Commun.

[23] Pazhanivel N, Saahithya R, Thangapandiyan M, Venkata G, Rao S, Sridhar K (2018). Pulmonary aspergillosis in a seventeen-day old ostrich chick (*Struthio camelus*). J. Entomol. Zool. Stud.

